# Rapid isolation method for extracellular vesicles based on Fe_3_O_4_@ZrO_2_

**DOI:** 10.3389/fbioe.2024.1399689

**Published:** 2024-07-09

**Authors:** Cuidie Ma, Zhihui Xu, Kun Hao, Lingling Fan, Wenqian Du, Zhan Gao, Chong Wang, Zheng Zhang, Ningxia Li, Qi Li, Qi Gao, Changyuan Yu

**Affiliations:** ^1^ College of Life Science and Technology, Beijing University of Chemical Technology, Beijing, China; ^2^ Beijing Hotgen Biotech Co., Ltd., Beijing, China; ^3^ Department of Urology, Xiyuan Hospital, China Academy of Chinese Medical Sciences, Beijing, China; ^4^ Department of Clinical Laboratory, The Second Hospital of Hebei Medical University, Shijiazhuang, Hebei, China; ^5^ Department of Clinical Laboratory, The First Hospital of Hebei Medical University, Shijiazhuang, Hebei, China; ^6^ Department of Clinical Laboratory, The Second Affiliated Hospital of Xi’an Medical University, Xi’an, Shaanxi, China; ^7^ Department of Clinical Laboratory, Xiyuan Hospital, China Academy of Chinese Medical Sciences, Beijing, China

**Keywords:** extracellular vesicles, microRNAs, ZrO_2_, isolation, prostate cancer

## Abstract

Extracellular vesicles (EVs) are pivotal in intercellular communication, disease mechanisms. Despite numerous methods for EVs isolation, challenges persist in yield, purity, reproducibility, cost, time, and automation. We introduce a EVs isolation technique using Fe_3_O_4_@ZrO_2_ beads, leveraging ZrO_2_-phosphate interaction. The results indicated that EVs were efficiently separated from large volumes of samples in 30 minutes without preconcentration. Our method demonstrated capture efficiency (74%–78%) compared to ultracentrifugation, purity (97%), and reproducibility (0.3%–0.5%), with excellent linearity (R^2^ > 0.99). EVs from urine samples showed altered expression of miRNAs. The logistic regression model achieved an AUC of 0.961, sensitivity of 0.92, and specificity of 0.94. With potential for automation, this magnetic bead-based method holds promise for clinical applications, offering an efficient and reliable tool for EVs research and clinical studies.

## 1 Introduction

Extracellular vesicles (EVs) are nanoscale, membrane-bound vesicles secreted by various cells, serving as nanoscale messengers that encapsulate diverse biomolecules, including RNAs, lipids, and proteins (Alvarez-Erviti et al., 2011; Azmi et al., 2013; Jenjaroenpun et al., 2013; Cooper et al., 2014; Lobb et al., 2015). These vesicles are ubiquitous in various body fluids and tissues, such as blood, urine, ascites, and brain tissue, and play crucial roles in diseases pathogenesis and progression (Denzer et al., 2000; Schorey and Bhatnagar, 2008; Ogorevc et al., 2013; Xu et al., 2016; Vella et al., 2017). Reflecting the physiological and pathological status of their originating cells, EVs have been identified as potential biomarkers for cancers, metabolic and neurodegenerative disorders, as they can transport associated proteins, miRNAs, and other critical molecules (Farooqi et al., 2018; Akbar et al., 2019; Hill, 2019; Xia et al., 2022). Furthermore, EVs mediate intercellular communication, regulating the gene expression and cellular functions, which is instrumental in disease onset and progression, making them ideal tools for studying disease mechanisms and therapeutic target identification (Gatti et al., 2011).

In recent times, the analysis of EVs in urine has gained increasing attention, particularly for their potential in urinary diagnostics. Urine-derived EVs, characterized by their broad origin, easy accessibility, and non-invasive collection, present exciting prospects for early disease detection, especially for conditions like cancer and kidney diseases (Erdbrugger et al., 2021).

The quality of EV samples is greatly dependent on the chosen isolation method. The traditional methods for EV isolation are using ultracentrifugation or differential centrifugation, immunoaffinity based capture using specific membrane proteins such as CD9, CD63, and CD31, and size-based capture using size-exclusion chromatography (Szatanek et al., 2015; Jeppesen et al., 2019). In addition, alternative methods for EV isolation have been introduced, such as microfluid-based methods through viscoelastic flow, filtration, aptamer-mediated sorting, acoustic isolation, and polymer precipitation (Contreras-Naranjo et al., 2017; Liu et al., 2017; Liu et al., 2019). However, current techniques often face challenges like complex and time-consuming procedures, limited efficiency, and constraints in processing large volume of samples (Baranyai et al., 2015; Campoy et al., 2016; An et al., 2018; Dong et al., 2020). EV existence in a wide range of nanosize particles, at low concentrations and at high heterogeneities (Merchant et al., 2017; Zhang et al., 2021a; Zhang et al., 2021b). Specifically, the isolation of EVs from urine poses unique challenges due to the low concentration of EVs and the requirement for substantial sample volumes, which is impractical in cases of limited sample availability. These limitations impact the reliability and applicability of urine-derived EVs in clinical settings (Merchant et al., 2017), underscoring the need for more efficient and practical isolation techniques.

Metal oxides have attracted significant attention in the field of EVs isolation, due to their unique physical and chemical properties. Their ability to selectively bind to specific components on EV surfaces has been leveraged in various studies. For example, materials based on titanium dioxide (TiO_2_) have demonstrated high efficiency and purity in isolating EVs through interactions with phosphate groups (Gao et al., 2019; Zhang et al., 2021a; Zhang et al., 2021b; Zhang H. et al., 2021; Xiang et al., 2021; Ma et al., 2023). Qian et al. first developed a TiO_2_-based separation method for isolating serum EVs with high recovery rates, which could be characterized and analyzed for protein content (Gao et al., 2019). Qin et al. further proposed a strategy for isolating EVs from human urine using a method based on UC and TiO_2_, obtaining high-quality EVs suitable for LC-MS/MS analysis (Xiang et al., 2021). Additionally, Di et al. (2022) utilized Fe_3_O_4_@TiO_2_ beads for magnetic separation based on TiO_2_, enabling the direct heating and efficient release of target miRNAs from isolated EVs for subsequent analysis. These research advances open up new possibilities for the application of metal oxides in EVs isolation, providing more flexible and effective means for both research and clinical applications of EVs. Ti and Zr belong to the same chemical family, sharing identical outer electron shell configurations. This similarity determines their tendency to exhibit comparable chemical properties in reactions. Inspired by these findings, we explored the possibility of utilizing ZrO_2_ in a manner similar to TiO_2_, by employing phosphate groups for the extraction of extracellular vesicles. This research opens up new possibilities for the application of metal oxides in the extraction of extracellular vesicles, providing a more flexible and effective approach for both the study and clinical applications of extracellular vesicles.

MiRNAs, short RNA molecules approximately 20–24 nucleotides in length, plays a crucial role in regulating various biological processes, including cell differentiation, proliferation, apoptosis, and are intricately linked with the occurrence and development of diseases (O'Brien et al., 2018). EV-mediated miRNA transport plays a significant role in intercellular communication, disease diagnosis and therapy (Zhang et al., 2019). miR-21, commonly upregulated in several cancers, is a widely recognized cancer biomarker linked to critical processes like tumor cell proliferation and metastasis (Abue et al., 2015; Kang et al., 2015; Petrovic, 2016; Bica-Pop et al., 2018; Li et al., 2018; Stafford et al., 2022). Similarly, miR-125a in EVs can influence target cell behavior by regulating genes involved in tumor growth and migration (Babalola et al., 2013). miR-16 is often used as an internal reference gene in EV miRNAs studies for standardizing the expression levels of miRNA in EVs. These genes are generally considered to have minimal expression changes under different conditions, making them stable reference standards (Huang et al., 2010; Aalami et al., 2023).

In this study, we propose a novel, rapid method for EV isolation, utilizing Fe_3_O_4_ beads and ZrO_2_. This technique capitalizes on the interaction between ZrO_2_ and phosphate groups to efficiently separate EVs from large volumes of cell supernatants and urine samples without preconcentration. We assessed the capture efficiency through RT-qPCR and investigated the differential expression of EV miRNAs associated with prostate cancer in urine samples.

## 2 Materials and methods

### 2.1 Collection of samples

The urine samples were obtained from healthy volunteers and persons with prostate cancer at Xiyuan Hospital, China Academy of Chinese Medical Sciences. Inclusion criteria were age ≥18 years, and exclusion criteria included pregnancy, immunosuppression, other cancers, or initiation of palliative treatment. All procedures were reviewed and approved by the Ethics Committee of Xiyuan Hospital, China Academy of Chinese Medical Sciences (2020XLA067-2), in accordance with the Helsinki Declaration. The experiments were undertaken with the understanding and written consent of each subject. The clinical characteristics of the participants are presented in Table 1. Initially, on the next morning, midstream urine was collected in a 50 mL centrifuge tube, centrifuged at 2,000 *g* for 10 min at room temperature, and the supernatant was stored at −80°C and avoided repeated freeze-thaw cycles.

**TABLE 1:** The clinical characteristics of participants in this study.

Characteristics		Total (n=40)	
		Prostate cancer (n=24)	Healthy control (n=16)
Age	Mean ± SD	67.96±5.171	68.75±4.041
Sex	Female	0	0
	Male	24	16
BMI	Underweight	0	0
	Normal	10	7
	Overweight	13	9
	Obese	1	0
Drinking history	Yes	14	9
	No	10	7
Family history	Yes	5	0
	No	19	16
TNM	I	9	-
	II	7	-
	III	7	-
	Ⅳ	1	-

Human embryonic kidney cell line HEK293T was purchased from the Shanghai Cell Bank, Chinese Academy of Sciences (Shanghai, China). Cells were cultured in Dulbecco’s Modified Eagle Medium (DMEM, pH: 7.0–7.4, Thermo Fisher Scientific) containing 10% (v/v) fetal bovine serum (FBS, Thermo Fisher Scientific) at 5% CO_2_ on 37°C without any addition of antibiotics. In each passage, 2 × 10^6^ cells were seeded into 15 mL of cell medium in a culturing flask and cultured in an incubator (Panasonic Corp). Once 80% confluency was achieved, the supernatant was carefully removed and the cells were washed twice with PBS. Next, the cells were cultured in DMEM with 10% exosomes depleted FBS (System Bioscience) for 24 h and the culture medium was collected. Cell culture supernatant was centrifuged at 300 *g* for 10 min at room temperature to remove cell debris, and then at 2,000 g for 10 min at room temperature to collect the supernatant, which was stored at −80°C for later use and avoided repeated freeze-thaw cycles.

### 2.2 Isolation of EVs

UC: The cell culture supernatant was centrifuged at 2,000 *g* for 20 min at room temperature, and the supernatant was further centrifuged at 1,10,000 g for 2 h at 4°C (Beckman Coulter, Brea, CA, United States) to obtain the EVs. After discarding the supernatant, EVs were resuspended in 200 μL of PBS. The EVs was stored at −80°C and avoided repeated freeze-thaw cycles.

**FIGURE 1:**
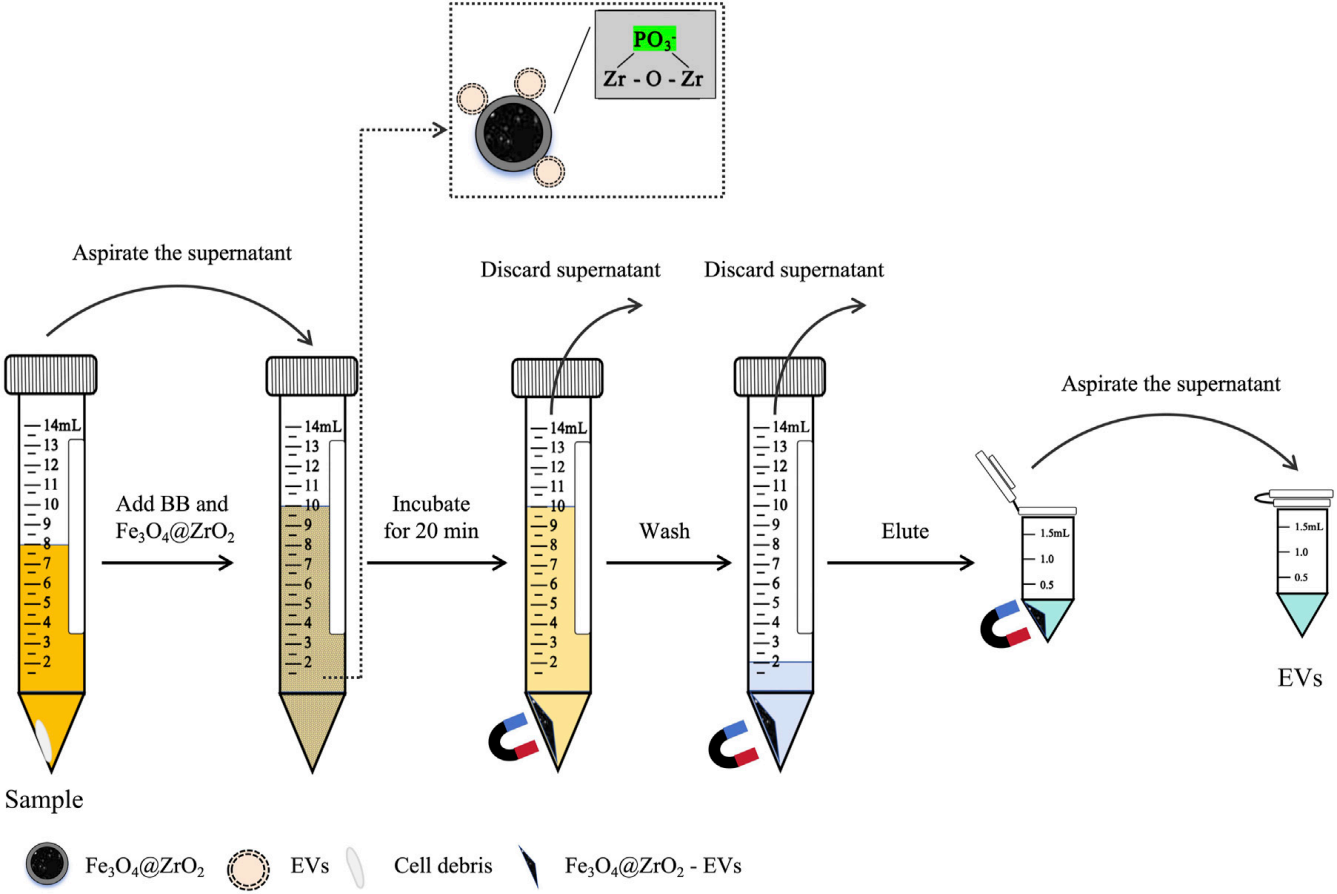
Diagram for capturing EVs based on Fe_3_O_4_@ZrO_2_. After centrifugation, the supernatant was collected, and binding buffer (BB) containing Fe_3_O_4_@ZrO_2_ beads was added to the supernatant. After incubation for 20 min, magnetic separation was performed to discard the supernatant. Wash buffer (WBF) was added to the Fe_3_O_4_@ZrO_2_ beads, washed twice, and the supernatant was discarded. Finally, elution buffer (EB) was added to the Fe_3_O_4_@ZrO_2_ beads for elution. After incubation for 5 min, magnetic separation was performed to collect the supernatant, which contains the EVs.

Fe_3_O_4_@ZrO_2_: Fe_3_O_4_@ZrO_2_ beads provided by Beijing Hotgen Biotech Co., Ltd. were added to binding buffer (BB, 0.5 M sodium acetate, pH adjusted to 5.5 with ice acetic acid). 8 mL sample was mixed with 2 mL of BB containing Fe_3_O_4_@ZrO_2_ beads (0.5 mg/mL) and incubated for 20 min at room temperature. After sufficient incubation, the magnetic bead-EV complexes were magnetically retained, and non-EV components were washed away using wash buffer (WBF, 0.007% cetyltrimethylammonium bromide). The EVs were then released from the magnetic beads using 200 μL of elution buffer (EB, 50 mM sodium carbonate, pH adjusted to 10.5 with sodium bicarbonate), as illustrated in Figure 1. The EVs was stored at −80°C and avoided repeated freeze-thaw cycles.

Fe_3_O_4_@TiO_2_: Fe_3_O_4_@TiO_2_ beads provided by Beijing Hotgen Biotech Co., Ltd. were added to binding buffer (BB, 0.5 M sodium acetate, pH adjusted to 5.5 with ice acetic acid). 8 mL sample was mixed with 2 mL of BB containing Fe_3_O_4_@TiO_2_ beads (0.5 mg/mL) and incubated for 20 min at room temperature. After sufficient incubation, the magnetic bead-EV complexes were magnetically retained, and non-EV components were washed away using wash buffer (WBF, 0.007% cetyltrimethylammonium bromide). The EVs were then released from the magnetic beads using 200 μL of elution buffer (EB, 50 mM sodium carbonate, pH adjusted to 10.5 with sodium bicarbonate). The EVs was stored at −80°C and avoided repeated freeze-thaw cycles.

### 2.3 Characterization of EVs

#### 2.3.1 Transmission electron microscopy (TEM)

EVs were fixed with 2% glutaraldehyde at room temperature for 1 h, followed by staining with uranyl acetate solution (pH 4.5). Images were observed under FEI Tecnai Spirit (FEI, Eindhoven, Netherlands) at 120 kV, and electron micrographs were captured using a Gatan UltraScan 1000 charge-coupled device (CCD) camera (Pleasanton, CA, United States).

#### 2.3.2 Nanoparticle tracking analysis (NTA)

EVs were diluted in PBS to a certain concentration. Nanoparticle tracking analysis was performed using ZetaView^®^ PMX120 (Particle Metrix GmbH, Meerbusch, Germany) to determine the size and quantity of separated particles. The collected data were analyzed using Software ZetaView (version 8.05.16 SP2) to study particle movement.

#### 2.3.3 Western blot analysis (WB)

Prior to Western blot analysis, EV samples were lysed by RIPA lysis buffer (Cat. 89900, Thermo Fisher Scientific, Loughborough, United Kingdom) on ice for 5 min, then centrifuged at 14,000 *g* for 15 min at room temperature to collect the cell debris. Protein concentration was determined using a Qubit Protein Assay Kit (Invitrogen, Pittsburgh, PA, United States) and a Qubit 4.0 Fluorometer (Invitrogen, Pittsburgh, PA, United States). 10 μg of total protein were loaded onto 10% SDS-PAGE Color Preparation kit (Cat. C671102, Sangon Biotech, Shanghai, China) for electrophoresis. Following electrophoresis, gels were transferred to 0.2 μm PVDF (polyvinylidene fluoride) membranes (Millipore, Billerica, United States) for Western blot ting. Membranes were blocked with 5% nonfat dry milk dissolved in 1 × PBST (PBS plus 0.2% v/v Tween-20) for 1 h at room temperature and washed with 1 × PBST solution three times. Each washing time is 10 min. Then the membranes were incubated with primary antibodies overnight at 4°C and washed with 1 × PBST solution three times. Each washing time is 10 min. Then the secondary antibody rabbit anti-mouse IgG-HRP (ab6728) (1:1000 dilution, Abcam, Cambridge, MA, United States) was incubated with the membranes at room temperature for 1 h. Primary antibodies included anti-CD9 (EPR23105-125) (1:1000 dilution, Abcam, Cambridge, MA, United States), anti-TSG101 (EPR7130(B)) (1:1000 dilution, Abcam, Cambridge, MA, United States), and anti-calnexin (EPR3633(2)) (1:1000 dilution, Abcam, Cambridge, MA, United States). The protein bands were detected using the BeyoECL Plus Detection System (Beyotime Biotechnology, Shanghai, China).

### 2.4 RNA isolation and analysis

#### 2.4.1 RNA isolation

Total RNA was extracted from the isolated EVs utilizing the miRNeasy^®^ Mini Kit (Cat. 217004, Qiagen, Hilden, Germany) following the recommended protocol provided by the manufacturer. Subsequently, the RNA concentration was determined using the Qubit microRNA Assay (Invitrogen, Pittsburgh, PA, United States) and the Qubit 4.0 Fluorometer (Invitrogen, Pittsburgh, PA, United States).

#### 2.4.2 RT-qPCR

A 10 μL mixture for the RT reaction was prepared, consisting of 2 μL 5× HiScript III Buffer, 0.5 μL HiScript III Reverse Transcriptase, 0.5 μL *E. coli* Poly(A) Polymerase, 1 μL ATP (10 mM), 1 μL Super pure dNTP (10 mM), 2 μL RT primer (10 μM), and 3 μL RNA. The RT reaction was conducted at 42°C for 60 min, followed by a 5 min incubation at 85°C.

qPCR (TaqMan) was performed on an ABI 7500 Real-Time PCR System (Applied Biosystems, Foster City, CA, United States). The 25 μL PCR mixture comprised 12.5 μL 2× Robustart Premix Omni III (Probe qPCR), 2 μL PCR forward primer (10 μM), 2 μL PCR reverse primer (10 μM), 1 μL PCR probe (10 μM), 0.5 μL 50× Rox reference dye, 2 μL ddH_2_O, and 5 μL cDNA (5-fold diluted). The reaction commenced with a 5 min incubation at 95°C, followed by 45 amplification cycles of 15 s at 95°C and 32 s at 60°C. The RT-qPCR primers used in this study were synthesized by Biotech (Shanghai, China) Co., Ltd. The sequences of primers and probes employed are presented in Supplementary Table S1.

### 2.5 Statistical analysis

The relative expression levels of miRNAs were determined using the 2^−ΔΔCT^ method. Statistical analysis was conducted using GraphPad Prism nine software (GraphPad Software, San Diego, CA, United States). Data were analyzed for statistical significance using appropriate tests, such as Student’s t-test, one-way analysis of variance (ANOVA), or two-way ANOVA, as applicable. Results were considered significantly altered when *p* < 0.05. To compare the performance of the models, we calculated the mean of the ROC curves and their 95% confidence intervals. This analysis was conducted using the “roc-utils” package in Python 3.11.3, with the aid of 1000 bootstrap samples. The optimal cutoff point was determined in accordance with the maximum Youden’s index principle. In order to assess the prediction model’s performance on the validation dataset using this optimal cutoff, we generated ROC curve with GraphPad Prism 9 software. The sensitivity and specificity were calculated using sklearn package in Python 3.11.3.

## 3 Results

### 3.1 Characterization of EVs based on Fe_3_O_4_@ZrO_2_ beads

**FIGURE 2:**
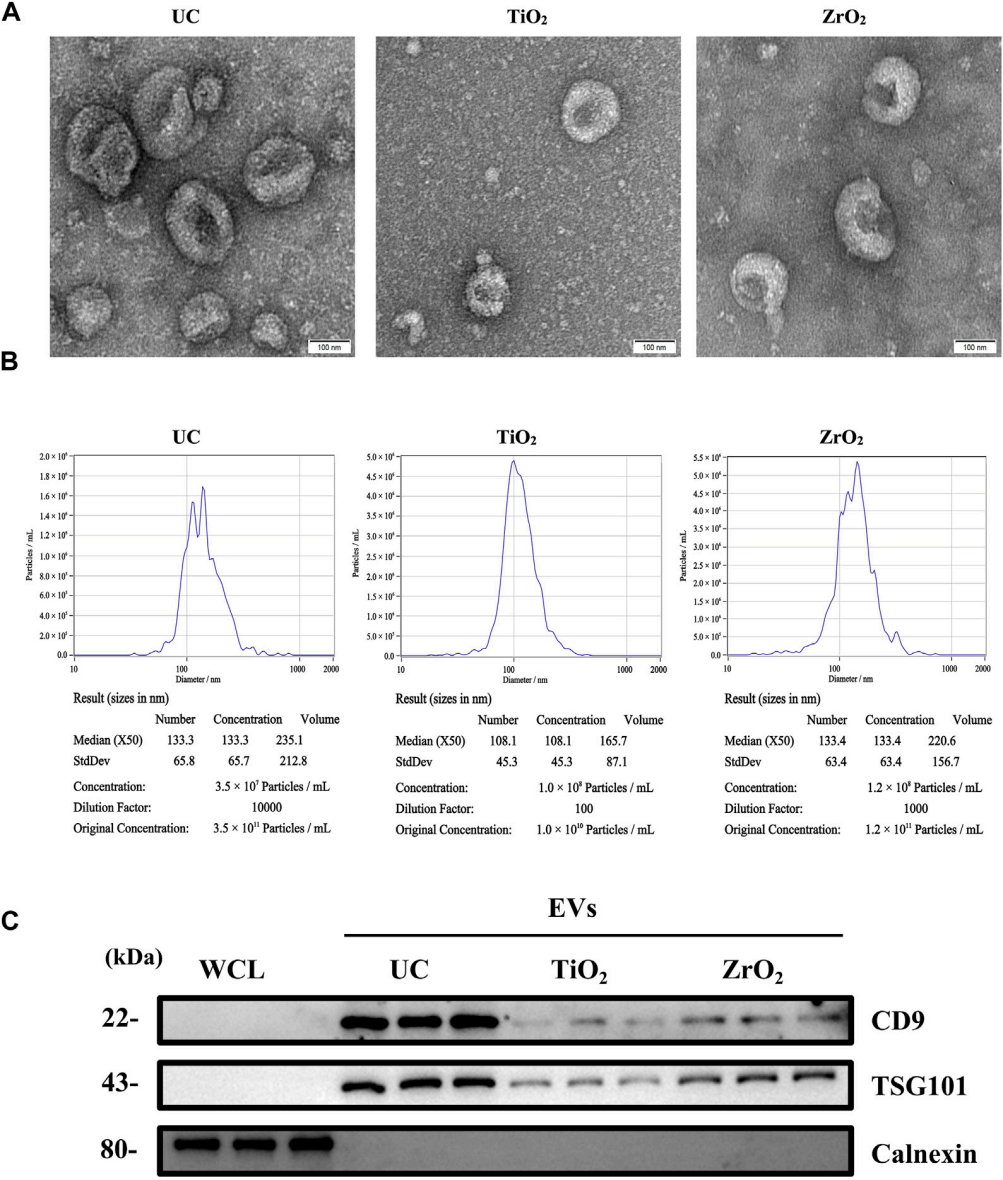
Characterization of EVs **(A)** Transmission electron microscopy (TEM) image of EVs isolated by ultracentrifugation (UC), Fe_3_O_4_@TiO_2_, Fe_3_O_4_@ZrO_2_ (bar = 100 nm) **(B)** Nanoparticle tracking analysis (NTA) of EVs isolated by UC, Fe_3_O_4_@TiO_2_, Fe_3_O_4_@ZrO_2_ **(C)** Western blot (WB) analysis of whole cell lysate (WCL), UC-EVs, Fe_3_O_4_@TiO_2_-EVs, Fe_3_O_4_@ZrO_2_-EVs. There are three replicates for each experimental condition.

In this study, to demonstrate that Fe_3_O_4_@ZrO_2_ beads can capture EVs, we characterized the EVs from cell culture supernatants captured by three methods: UC, as the gold standard, capture using Fe_3_O_4_@TiO_2_ beads (reported in the literature for EVs capture), and capture using Fe_3_O_4_@ZrO_2_ beads. TEM revealed cup-shaped structures in EVs isolated by all three methods, and NTA showed particle sizes of 149.1 ± 65.8 nm, 119.7 ± 45.3 nm, and 145.5 ± 63.4 nm for UC, Fe_3_O_4_@TiO_2_, and Fe_3_O_4_@ZrO_2_, respectively, consistent with typical EVs sizes (Figures 2A, B). WB analysis confirmed the presence of EV marker proteins CD9 and TSG101, with no detection of the negative control protein calnexin (Figure 2C). This evidence suggests that EVs isolated by Fe_3_O_4_@ZrO_2_ beads exhibit similar size and morphology to those obtained by the widely accepted UC method, successfully isolating EVs from cell culture supernatants.

### 3.2 Capture efficiency of Fe_3_O_4_@ZrO_2_ beads for EVs

**FIGURE 3:**
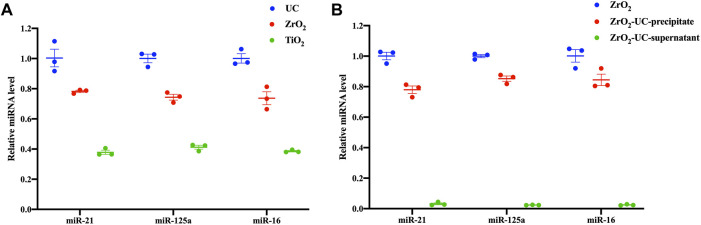
The capture efficiency of capturing EVs based on Fe_3_O_4_@ZrO_2_ **(A)** Relative expression levels of three EV miRNAs isolated by ultracentrifugation (UC), Fe_3_O_4_@TiO_2_(TiO_2_), Fe_3_O_4_@ZrO_2_ (ZrO_2_) **(B)** Relative expression levels of three EV miRNAs in three groups: The EV miRNAs were isolated by Fe_3_O_4_@ZrO_2_(ZrO_2_). The elution buffer obtained based on Fe_3_O_4_@ZrO_2_ was subjected to UC, and EV miRNAs was extracted from the precipitate (ZrO_2_-UC-precipitate). The elution buffer obtained based on Fe_3_O_4_@ZrO_2_ was subjected to UC, and EV miRNAs was extracted from the supernatant (ZrO_2_-UC-supernatant). All data are represented as mean ± SEM.

After establishing the similarity in size and morphology between EVs isolated by Fe_3_O_4_@ZrO_2_ and UC-extracted EVs, it is crucial to assess the capture efficiency of this method. We measured the EV miRNAs, including miR-21, miR-125a, and miR-16, extracted by the three isolation methods (Figure 3A). There are three replicates for each experimental condition. Detailed statistical analysis information is provided in Supplementary Table S2. As shown in Table 2, miR-21 obtained with Fe_3_O_4_@ZrO_2_ beads was 78% of that obtained by UC, while miR-125a and miR-16 were both 74% of UC-extracted levels. In contrast, miR-21, miR-125a, and miR-16 obtained with Fe_3_O_4_@TiO_2_ beads were 38%, 41%, and 39% of UC levels, respectively. Overall, the isolation efficiency of Fe_3_O_4_@ZrO_2_ beads was higher compared to Fe_3_O_4_@TiO_2_ beads and comparable to UC.

**TABLE 2:** The isolation efficiency of EVs captured by Fe_3_O_4_@ZrO_2_ and Fe_3_O_4_@TiO_2_ compared to UC.

	miR-21	miR-125a	miR-16
UC	100.0%	100.0%	100.0%
ZrO_2_	78.0%	74.3%	73.6%
TiO_2_	37.7%	41.2%	38.5%

Furthermore, we demonstrated the purity of EVs captured by Fe_3_O_4_@ZrO_2_ beads. Using Fe_3_O_4_@ZrO_2_ beads to capture cell culture supernatants, the eluate was re-extracted using UC. RNA was then extracted from the pre-centrifugation eluate (ZrO_2_), post-UC pellet (ZrO_2_-UC-precipitate), and supernatant (ZrO_2_-UC- supernatant), followed by RT-qPCR. There are three replicates for each experimental condition. Using EVs in the eluate before UC as a control, the post-UC pellet showed miR-21, miR-125a, and miR-16 levels at 78%, 85%, and 84%, respectively, while the post-UC supernatant had levels of 3%, 2%, and 2%, as shown in Figure 3B; Table 3. Although the expression levels of EV miRNAs in the precipitate after UC were slightly reduced compared to before UC (78%–85%), we observed that EV miRNAs in the supernatant after UC accounted for only 2%–3% of the total expression. The results indicated a high purity level (up to 97%), with only minor differences (0.2–0.4) in CT values, despite a slight reduction in EV miRNA expression levels post-UC. This suggests that our method is effective, albeit with some loss likely due to procedural or experimental errors. Detailed statistical analysis information is provided in Supplementary Table S3. This evidence indicates that Fe_3_O_4_@ZrO_2_ beads can extract highly pure EVs from cell culture supernatants.

**TABLE 3:** The purity of EVs captured by Fe_3_O_4_@ZrO_2_ compared to UC.

	miR-21	miR-125a	miR-16
ZrO_2_	100.0%	100.0%	100.0%
ZrO_2_-UC-precipitate	78%	85%	84%
ZrO_2_-UC-supernatant	3%	2%	2%
Loss	19%	12%	13%
Purity	96%	97%	97%

### 3.3 Optimization of capture conditions

**FIGURE 4:**
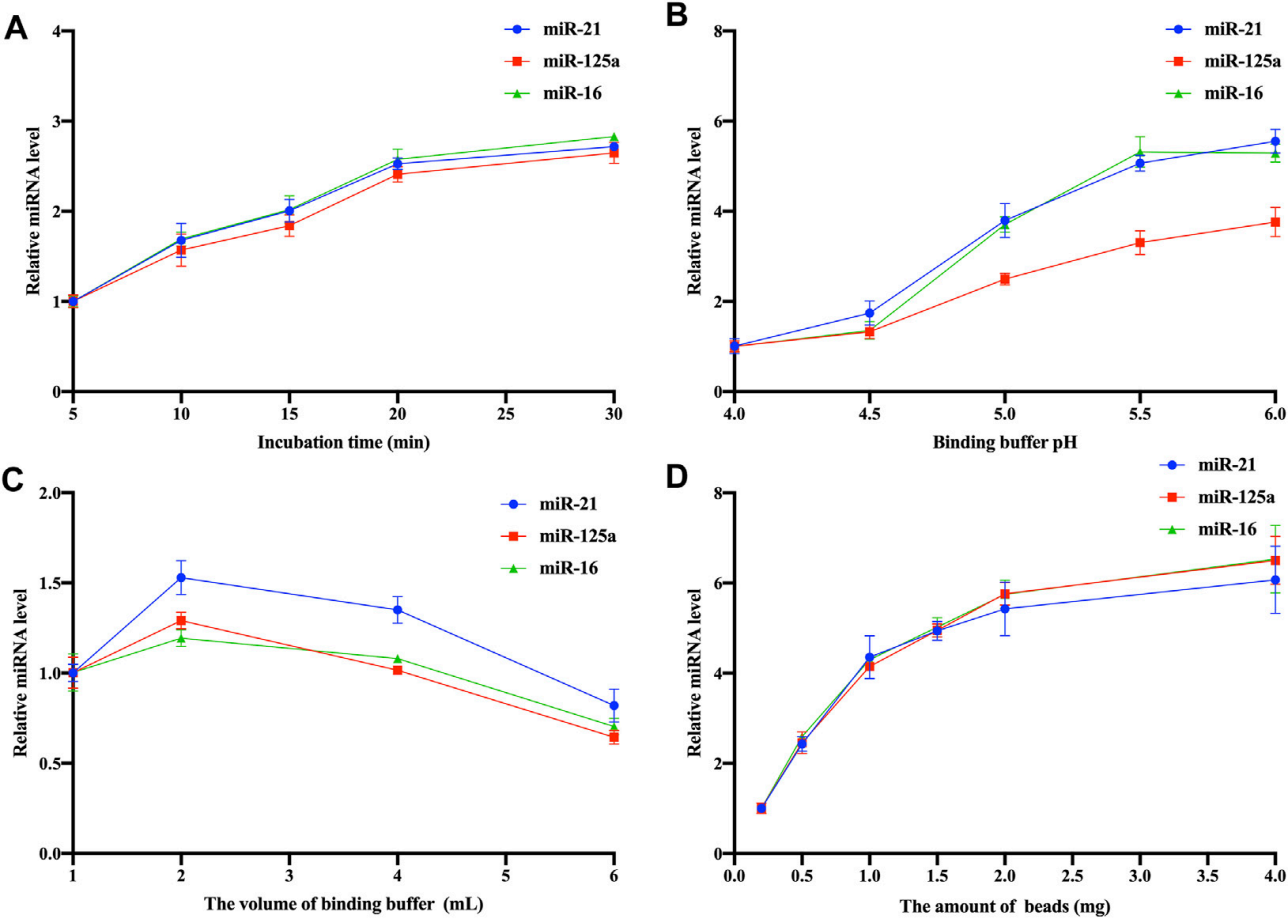
Optimization of Fe_3_O_4_@ZrO_2_ capture conditions **(A)** Optimization of incubation time **(B)** Optimization of binding buffer pH **(C)** Optimization of binding buffer volume **(D)** Optimization of bead amount. All data are represented as mean ± SD.

To enhance capture efficiency, optimization experiments were conducted, focusing on incubation time, pH of the BB, volume of the binding buffer, and amount of Fe_3_O_4_@ZrO_2_ beads. The relative expression levels of captured EV miRNAs were calculated using the 2^−ΔΔCT^ method, with each group’s initial values as controls. There are three replicates for each experimental condition. Detailed statistical analysis information is provided in Supplementary Tables S4–S7. As shown in Figure 4A, the relative expression of captured EV miRNAs increased with prolonged incubation time, reaching optimal levels at 20 min. Although the 30-min curve showed a slight increase in miRNA relative expression, the difference was not statistically significant. pH of the BB influenced the capture process, with optimal expression achieved at pH 5.5 (Figure 4B). The optimal volume of BB was 2 mL (Figure 4C). Increasing the amount of Fe_3_O_4_@ZrO_2_ beads from 0.2 mg to 4 mg enhanced the relative expression of captured EV miRNAs. However, there were no significant differences among experimental groups beyond 1 mg, making 1 mg the most suitable quantity (Figure 4D).

### 3.4 Performance validation of the EVs capture method based on Fe_3_O_4_@ZrO_2_

**FIGURE 5:**
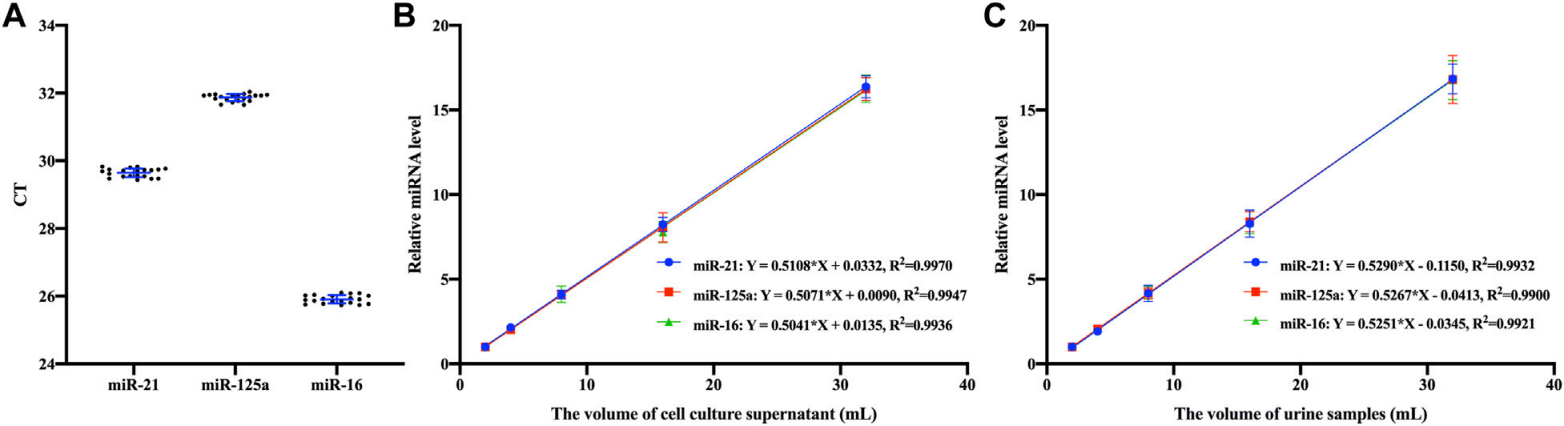
Assay features validation **(A)** Repeated experimental results of three EV miRNAs based on Fe_3_O_4_@ZrO_2_ in cell culture supernatant **(B)** Linear fitting between relative expression levels of three EV miRNAs based on Fe_3_O_4_@ZrO_2_ and the volume of cell culture supernatant **(C)** Linear fitting between relative expression levels of three EV miRNAs based on Fe_3_O_4_@ZrO_2_ and the volume of urine samples. All data are represented as mean ± SD.

After optimizing the capture conditions and establishing the isolation system, the reproducibility and linearity of the EVs capture method based on Fe_3_O_4_@ZrO_2_ were verified. As shown in Figure 5A, the method demonstrated excellent reproducibility, with CV of 0.4%, 0.3%, and 0.5% for ten replicates of miR-21, miR-125a, and miR-16, respectively. The linear relationship between sample volume and relative expression of EV miRNAs was tested separately for cell culture supernatant and urine samples using Fe_3_O_4_@ZrO_2_ beads. There are three replicates for each experimental condition. Equations and *R* ^2^ values are presented in Figures 5B, C, showing a linear relationship between sample volumes of 2–32 mL for both cell culture supernatant and urine samples.

### 3.5 Application of the capture method based on Fe_3_O_4_@ZrO_2_ in clinical samples

**FIGURE 6:**
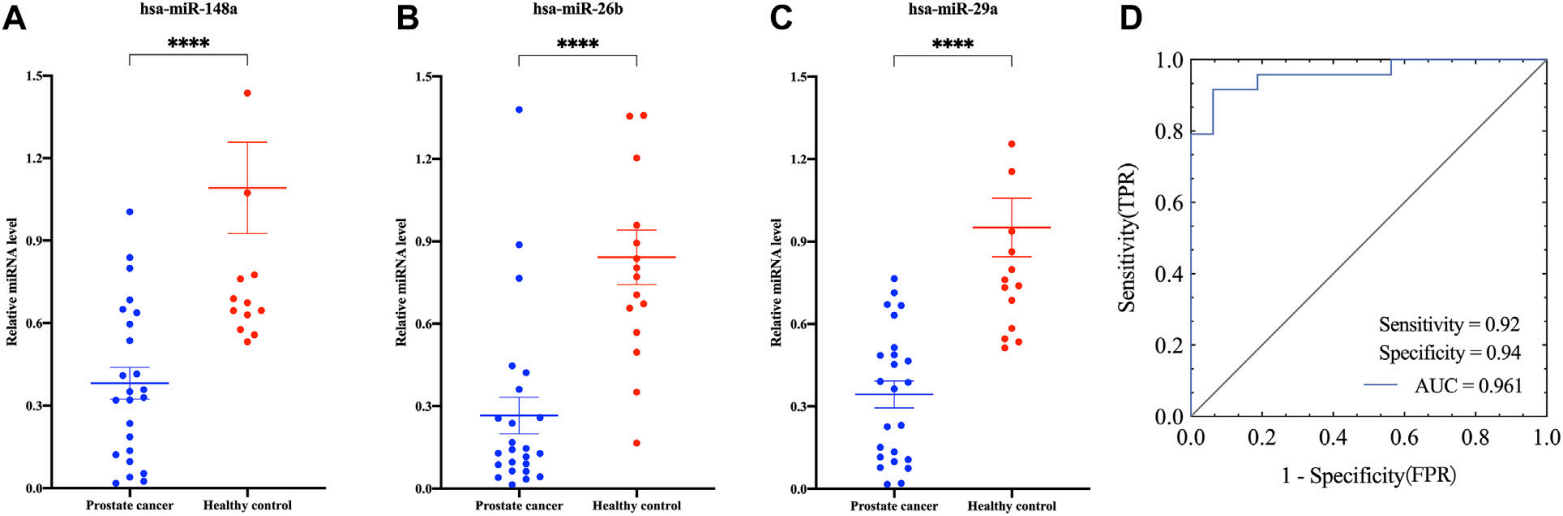
Application of the capture method based on Fe_3_O_4_@ZrO_2_ in Clinical Samples. **(A)** The relative expression levels of hsa-miR-148a in EVs associated with prostate cancer based on Fe_3_O_4_@ZrO_2_ in persons with prostate cancer and healthy controls. **(B)** The relative expression levels of hsa-miR-26b in EVs associated with prostate cancer based on Fe_3_O_4_@ZrO_2_ in persons with prostate cancer and healthy controls. **(C)** The relative expression levels of hsa-miR-29a in EVs associated with prostate cancer based on Fe_3_O_4_@ZrO_2_ in persons with prostate cancer and healthy controls. **(D)** ROC curve of a model established by EV miRNAs associated with prostate cancer. ****p < 0. 0001. All data are represented as mean ± SEM.

To further explore the application of this isolation method in clinical biomarkers, EVs were isolated from urine samples of healthy donors (n = 16) and persons with prostate cancer (n = 24) using Fe_3_O_4_@ZrO_2_ beads. Using miR-16 as an internal reference, the relative expression levels of hsa-miR-148a, hsa-miR-26b, and hsa-miR-29a, reported in the literature to be associated with prostate cancer, were calculated. As shown in Figures 6A-D, the expression levels of hsa-miR-148a, hsa-miR-26b, and hsa-miR-29a were all downregulated. The logistic regression model established using these miRNAs showed an AUC of 0.96, sensitivity of 0.92, and specificity of 0.94 with a cut off value set at 0.48. The logistic regression equation is shown in Formulas 1, 2:

$$z=0.97\times {∆Ct}_{\left(miR-148a-miR-16\right)}+0.77\times {∆Ct}_{\left(miR-26b-miR-16\right)}+0.53\times {∆Ct}_{\left(miR-29a-miR-16\right)}$$ (1)

$$y=\frac{1}{1+e^{-z}}$$ (2)

## 4 Discussion

EVs are increasingly recognized as carriers and mediators of intercellular communication. Serving as natural biological nanoparticles, EVs can be secreted by a diverse range of cell types and found in various biological fluids. While numerous methods for isolating EVs have been explored over the past decades, they often face challenges such as limited yield and purity, reproducibility issues, high costs, time-intensive processes, and difficulties in automation. The normative limitations of traditional methods have exacerbated the demand for better enrichment strategies (Holcar et al., 2021). Advanced methods, such as the acoustic fluid method, generate EVs equivalent to high-speed centrifugation by using very small sample volumes, but there is still a need to study optimized combinations of acoustic/microfluidic forces to effectively control small and buoyant nanoparticle EVs (Gu et al., 2021). For low concentration, large volume samples such as urine, the operation is cumbersome or requires preconcentration.

In this study, we developed a novel, efficient, and cost-effective technique for EVs isolation using Fe_3_O_4_@ZrO_2_ beads. The technique utilizes the interaction between ZrO_2_ and phosphate groups for EV separation from large volumes of cell culture supernatants and urine samples. Our method also demonstrated reproducibility and excellent linearity within a clinically relevant range of sample volumes (2–32 mL). This adaptability to varying sample sizes enhances its practicality for clinical applications.

In recent years, significant progress has been made in the development of EVs enrichment and detection platforms based on magnetic beads, which have been favored for their simplicity, robustness, and cost-effectiveness (Martín-Gracia et al., 2020). Recently, research has developed Fe_3_O_4_@TiO_2_ coated with titanium oxide for enriching EVs from serum. Fe_3_O_4_@TiO_2_ is based on selective EV concentration, which is fast and efficient. Fe_3_O_4_@TiO_2_ beads greatly reduce the isolation time of EV and reduce the loss of EV during the concentration step without affecting its proteome (Gao et al., 2019; Zhang et al., 2021a). In comparing the performance of the Fe_3_O_4_@ZrO_2_ technique with the established Fe_3_O_4_@TiO_2_ method, we observed similarities in operation principles and EV sizes captured (Di et al., 2022). Notably, our results suggest a higher binding efficiency of ZrO_2_ to phosphate groups compared to TiO_2_. However, this observation may be influenced by experimental conditions, sample properties, and detection methods. Further research will contribute to a more in-depth understanding of the performance differences between these two materials in phosphate group binding.

In clinical validation, our method successfully detected downregulated expressions of hsa-miR-148a, hsa-miR-26b, and hsa-miR-29a in urine samples from persons with prostate cancer, consistent with previous literatures (Nishikawa et al., 2014; Kato et al., 2015; Sengupta et al., 2018). Our study emphasizes the potential application of the developed method in non-invasive prostate cancer diagnosis. Using magnetic beads as a medium, it can be automated and has certain applicability in large-scale clinical trials, which is crucial for early diagnosis, monitoring treatment efficacy, and prognosis assessment, holding significant clinical significance.

Despite achieving promising results, our study has certain limitations that need careful consideration and resolution in future research. The specificity of the interaction between ZrO_2_ and phosphate groups central to our method may be influenced in different biological samples and environments. Future research should focus on validating this technique across a broader spectrum of sample types, including blood and tissue samples, to confirm its universal applicability.

In conclusion, our novel EVs isolation method shows significant promise. Its potential for automation, combined with its efficiency, cost-effectiveness, and speed, sets the stage for advancing EVs research and its applications in various biomedical fields.

## Data Availability

The original contributions presented in the study are included in the article/Supplementary Material, further inquiries can be directed to the corresponding authors.
