# Supplementary material for: Rapid isolation method for extracellular vesicles based on Fe3O4@ZrO2
Source: Front Bioeng Biotechnol. 2024 Jul 9;12:1399689. doi: 10.3389/fbioe.2024.1399689 (PMC11263208; doi:10.3389/fbioe.2024.1399689)
Supplement: Supplementary file 1 [file DataSheet1.PDF]

Supplementary Material

1 Supplementary Data

Table S1 The sequence of primers and probes.

Name	Sequence
RT primer	CGACTCGATCCAGTCTCAGGGTCCGAGGTAT TCGATCCTAACCCTCTCCTCGGTATCGAGTC GCACTTTTTTTTTTTTTTTVN
Forward primer-miR-21	GCGCTAGCTTATCAGACTGATGTT
Forward primer-miR-16	GCCCGTAGCAGCACGTAAATATT
Forward primer-miR-125a	GCGCTCCCTGAGACCCTTT
Forward primer-miR-148a	GCGCTCAGTGCACTACAGAACT
Forward primer-miR-26b	GCGCTTCAAGTAATTCAGGATAGGT
Forward primer-miR-29a	GTAGCACCATCTGAAATCGGTTA
Reverse primer	CAGTCTCAGGGTCCGAGGTATTC
Probe	TCGGTATCGAGTCGCACT

Table S2 Statistical analysis information of three EV miRNAs isolated by ultracentrifugation (UC), Fe_3O_4@TiO_2, Fe_3O4@ZrO_2.

miRNAs	Comparisons	Mean 1	Mean 2	Mean Diff.	95.00% CI of diff.	Below threshold?	Summary	Adjusted P Value
miR-21	UC vs. ZrO_2	1.0030	0.7823	0.2210	0.1147 to 0.3273	Yes	***	0.0001
	UC vs. TiO_2	1.0030	0.3783	0.6250	0.5186 to 0.7313	Yes	****	<0.0001
	ZrO_2 vs. TiO_2	0.7823	0.3783	0.4040	0.2976 to 0.5103	Yes	****	<0.0001
miR-125a	UC vs. ZrO_2	1.0010	0.7437	0.2571	0.1508 to 0.3634	Yes	****	<0.0001
	UC vs. TiO_2	1.0010	0.4121	0.5887	0.4823 to 0.6950	Yes	****	<0.0001
	ZrO_2 vs. TiO_2	0.7437	0.4121	0.3316	0.2252 to 0.4379	Yes	****	<0.0001
miR-16	UC vs. ZrO_2	1.0010	0.7371	0.2638	0.1575 to 0.3701	Yes	****	<0.0001
	UC vs. TiO_2	1.0010	0.3857	0.6153	0.5089 to 0.7216	Yes	****	<0.0001
	ZrO_2 vs. TiO_2	0.7371	0.3857	0.3515	0.2451 to 0.4578	Yes	****	<0.0001

***$P < 0.001$, ****$P < 0.0001$.

Table S3 Statistical analysis information of three EV miRNAs in three groups, including ZrO_2, ZrO_2-UC-precipitate, ZrO_2-UC-supernatant.

miRNAs	Comparisons	Mean 1	Mean 2	Mean Diff.	95.00% CI of diff.	Below threshold?	Summary	Adjusted P Value
miR-21	ZrO_2 vs. ZrO_2-UC-precipitate	1.0010	0.7795	0.2211	0.1379 to 0.3043	Yes	****	<0.0001
	ZrO_2 vs. ZrO_2-UC-supernatant	1.0010	0.0316	0.9690	0.8858 to 1.052	Yes	****	<0.0001
	ZrO_2-UC-precipitate vs. ZrO_2-UC-supernatant	0.7795	0.0316	0.7479	0.6647 to 0.8311	Yes	****	<0.0001
miR-125a	ZrO_2 vs. ZrO_2-UC-precipitate	1.0000	0.8524	0.1478	0.06455 to 0.2310	Yes	***	0.0007
	ZrO_2 vs. ZrO_2-UC-supernatant	1.0000	0.0238	0.9763	0.8931 to 1.060	Yes	****	<0.0001
	ZrO_2-UC-precipitate vs. ZrO_2-UC-supernatant	0.8524	0.0238	0.8285	0.7453 to 0.9117	Yes	****	<0.0001
miR-16	ZrO_2 vs. ZrO_2-UC-precipitate	1.0020	0.8447	0.1570	0.07379 to 0.2402	Yes	***	0.0004
	ZrO_2 vs. ZrO_2-UC-supernatant	1.0020	0.0245	0.9772	0.8940 to 1.060	Yes	****	<0.0001
	ZrO_2-UC-precipitate vs. ZrO_2-UC-supernatant	0.8447	0.0245	0.8202	0.7370 to 0.9034	Yes	****	<0.0001

***$P < 0.001$, ****$P < 0.0001$.

Table S4 Statistical analysis information of three EV miRNAs in different incubation time.

miRNAs	Comparisons	Mean 1	Mean 2	Mean Diff.	95.00% CI of diff.	Below threshold?	Summary	Adjusted P Value
miR-21	5 min vs. 10 min	1.0000	1.6790	-0.6784	-0.9340 to -0.4227	Yes	****	<0.0001
	10 min vs. 15 min	1.6790	2.0060	-0.3271	-0.5827 to -0.07139	Yes	**	0.007
	15 min vs. 20 min	2.0060	2.5270	-0.5211	-0.7768 to -0.2655	Yes	****	<0.0001
	20 min vs. 30 min	2.5270	2.7180	-0.1910	-0.4466 to 0.06471	No	ns	0.2199
miR-125a	5 min vs. 10 min	1.0020	1.5710	-0.5694	-0.8251 to -0.3137	Yes	****	<0.0001
	10 min vs. 15 min	1.5710	1.8400	-0.2694	-0.5251 to -0.01373	Yes	*	0.035
	15 min vs. 20 min	1.8400	2.4110	-0.5706	-0.8263 to -0.3150	Yes	****	<0.0001
	20 min vs. 30 min	2.4110	2.6490	-0.2375	-0.4931 to 0.01821	No	ns	0.0787
miR-16	5 min vs. 10 min	1.0010	1.6920	-0.6910	-0.9466 to -0.4353	Yes	****	<0.0001
	10 min vs. 15 min	1.6920	2.0200	-0.3273	-0.5830 to -0.07163	Yes	**	0.0069
	15 min vs. 20 min	2.0200	2.5760	-0.5561	-0.8117 to -0.3004	Yes	****	<0.0001
	20 min vs. 30 min	2.5760	2.8310	-0.2554	-0.5111 to 0.0002276	No	ns	0.0503

*P < 0.05, **P < 0.01, ****P < 0.0001, ns: nonsignificant.

Table S5 Statistical analysis information of three EV miRNAs in different binding buffer pH.

miRNAs	Comparisons	Mean 1	Mean 2	Mean Diff.	95.00% CI of diff.	Below threshold?	Summary	Adjusted P Value
miR-21	pH 4 vs. pH 4.5	1.0090	1.7410	-0.7320	-1.277 to -0.1869	Yes	**	0.0043
	pH 4.5 vs. pH 5	1.7410	3.7950	-2.0540	-2.599 to -1.509	Yes	****	<0.0001
	pH 5 vs. pH 5.5	3.7950	5.0680	-1.2730	-1.818 to -0.7280	Yes	****	<0.0001
	pH 5.5 vs. pH 6	5.0680	5.5550	-0.4865	-1.032 to 0.05868	No	ns	0.0982
miR-125a	pH 4 vs. pH 4.5	1.0050	1.3290	-0.3242	-0.8693 to 0.2210	No	ns	0.4348
	pH 4.5 vs. pH 5	1.3290	2.4980	-1.1690	-1.714 to -0.6236	Yes	****	<0.0001
	pH 5 vs. pH 5.5	2.4980	3.3060	-0.8083	-1.353 to -0.2631	Yes	**	0.0015
	pH 5.5 vs. pH 6	3.3060	3.7650	-0.4586	-1.004 to 0.08661	No	ns	0.1324
miR-16	pH 4 vs. pH 4.5	1.0000	1.3580	-0.3579	-0.9031 to 0.1872	No	ns	0.3369
	pH 4.5 vs. pH 5	1.3580	3.7070	-2.3480	-2.894 to -1.803	Yes	****	<0.0001
	pH 5 vs. pH 5.5	3.7070	5.3190	-1.6120	-2.157 to -1.067	Yes	****	<0.0001
	pH 5.5 vs. pH 6	5.3190	5.2920	0.0266	-0.5186 to 0.5717	No	ns	>0.9999

**P < 0.01, ****P < 0.0001, ns: nonsignificant.

Table S6 Statistical analysis information of three EV miRNAs in different binding buffer volumes.

miRNAs	Comparisons	Mean 1	Mean 2	Mean Diff.	95.00% CI of diff.	Below threshold?	Summary	Adjusted P Value
miR-21	1 mL vs. 2 mL	1.0010	1.5290	-0.5286	-0.6759 to -0.3812	Yes	****	<0.0001
	2 mL vs. 4 mL	1.5290	1.3510	0.1786	0.03127 to 0.3260	Yes	*	0.0134
	4 mL vs. 6 mL	1.3510	0.8203	0.5304	0.3830 to 0.6778	Yes	****	<0.0001
miR-125a	1 mL vs. 2 mL	1.0020	1.2910	-0.2884	-0.4358 to -0.1410	Yes	****	<0.0001
	2 mL vs. 4 mL	1.2910	1.0160	0.2746	0.1272 to 0.4220	Yes	***	0.0002
	4 mL vs. 6 mL	1.0160	0.6447	0.3716	0.2243 to 0.5190	Yes	****	<0.0001
miR-16	1 mL vs. 2 mL	1.0040	1.1940	-0.1906	-0.3380 to -0.04327	Yes	**	0.0079
	2 mL vs. 4 mL	1.1940	1.0800	0.1142	-0.03315 to 0.2616	No	ns	0.1699
	4 mL vs. 6 mL	1.0800	0.7035	0.3764	0.2291 to 0.5238	Yes	****	<0.0001

*P < 0.05, **P < 0.01, ***P < 0.001, ****P < 0.0001, ns: nonsignificant.

Table S7 Statistical analysis information of three EV miRNAs in different bead amount.

miRNAs	Comparisons	Mean 1	Mean 2	Mean Diff.	95.00% CI of diff.	Below threshold?	Summary	Adjusted P Value
miR-21	0.2 mg vs. 0.5 mg	1.0010	2.4280	-1.4260	-2.321 to -0.5315	Yes	***	0.0004
	0.5 mg vs. 1 mg	2.4280	4.3550	-1.9270	-2.822 to -1.032	Yes	****	<0.0001
	1 mg vs. 1.5 mg	4.3550	4.9410	-0.5859	-1.481 to 0.3088	No	ns	0.3787
	1.5 mg vs. 2 mg	4.9410	5.4270	-0.4862	-1.381 to 0.4085	No	ns	0.5817
	2 mg vs. 4 mg	5.4270	6.0730	-0.6459	-1.541 to 0.2488	No	ns	0.2755
miR-125a	0.2 mg vs. 0.5 mg	1.0040	2.4600	-1.4560	-2.351 to -0.5613	Yes	***	0.0003
	0.5 mg vs. 1 mg	2.4600	4.1500	-1.6900	-2.584 to -0.7948	Yes	****	<0.0001
	1 mg vs. 1.5 mg	4.1500	4.9470	-0.7972	-1.692 to 0.09751	No	ns	0.1042
	1.5 mg vs. 2 mg	4.9470	5.7630	-0.8155	-1.710 to 0.07918	No	ns	0.0913
	2 mg vs. 4 mg	5.7630	6.5030	-0.7400	-1.635 to 0.1547	No	ns	0.1545
miR-16	0.2 mg vs. 0.5 mg	1.0020	2.5950	-1.5930	-2.488 to -0.6985	Yes	****	<0.0001
	0.5 mg vs. 1 mg	2.5950	4.3020	-1.7070	-2.602 to -0.8125	Yes	****	<0.0001
	1 mg vs. 1.5 mg	4.3020	5.0190	-0.7167	-1.611 to 0.1780	No	ns	0.1798
	1.5 mg vs. 2 mg	5.0190	5.7500	-0.7311	-1.626 to 0.1636	No	ns	0.1638
	2 mg vs. 4 mg	5.7500	6.5320	-0.7813	-1.676 to 0.1134	No	ns	0.1167

***P < 0.001, ****P < 0.0001, ns: nonsignificant.
